# Investigation of the anticorrosion and adsorption properties of two polymer compounds on the corrosion of SABIC iron in 1 M HCl solution by practical and computational approaches

**DOI:** 10.1039/d2ra03614b

**Published:** 2022-07-12

**Authors:** M. Abdallah, K. A. Soliman, Mubark Alshareef, Arej S. Al-Gorair, H. Hawsawi, Hatem M. Altass, Salih S. Al-Juaid, M. S. Motawea

**Affiliations:** Chem. Depart., Faculty of Appl. Sci., Umm Al-Qura University Makkah Saudi Arabia metwally555@yahoo.com maabdelsaid@uqu.edu.sa; Chem. Depart., Faculty of Sci., Benha University Benha Egypt; Chem. Depart., College of Sci, Princess Nourah Bint Abdulrahman University Riyadh Saudi Arabia; University College of Alwajh, Tabuk University Alwajh Tabuk Saudi Arabia; Chem. Depart., Faculty of Sci., King Abdulaziz University Jeddah Saudi Arabia; Chem. Depart., Faculty of Sci., Tabuk University Tabuk Saudi Arabia

## Abstract

The anticorrosion efficiency of two polymer compounds, namely polystyrene (PS), polybutylene terephthalate (PBT), against the corrosion of SABIC iron (S-Fe) in 1.0 M HCl solution was investigated. The anticorrosion efficiency was estimated by chemical and electrochemical measurements. The anticorrosion efficiency increased with the increase in the concentration of the polymer compounds and reduction in temperature. All the obtained corrosion data confirmed the anticorrosion strength in the presence of PS and PBT compounds, such as the decreasing values of the corrosion current density, capacity of the double layer, and weight reduction, while the values of the charge-transfer resistance increased. Also, the pitting potential values moved in the noble (+) direction. The anticorrosion efficiency of the PBT compound was higher than that of the PS compound, which was 95.98% at 500 ppm concentration for PBT while for PS it was 93.34% according to polarization measurements. The anticorrosion activity occurred by the adsorption of PS and PBT compounds on the surface of S-Fe according to the Langmuir isotherm. The polarization curves indicated that the PS and PBT compounds were mixed-type inhibitors. Density functional theory (DFT) and Monte Carlo simulation (MC) were performed for the two polymer compounds. The computational quantum functions were found to be in agreement with the experimental results.

## Abstract 2

Top and side views for adsorption of the two dimers over Fe (110) surface.

## Graphical abstract

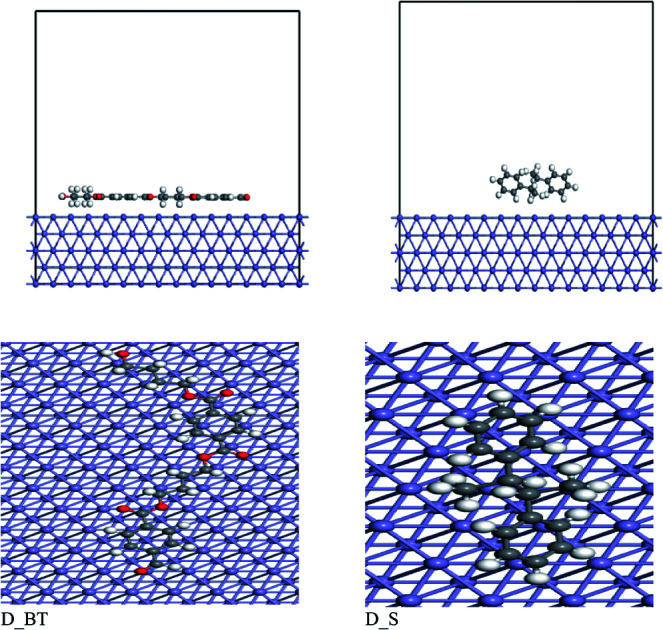

## 1. Introduction

SABIC iron (S-Fe) is considered one of the most important metals applied in industry, especially in the Kingdom of Saudi Arabia, where it is used in many vital industries, especially in construction.^1,2^ HCl solutions are applied for pickling, cleaning, and chemical and electrochemical etching of S-Fe. However, one of the disadvantages of using this acid is that it corrodes S-Fe. In order to diminish the risk of corrosion, scientists have turned to the use of corrosion inhibitors to diminish the rate of corrosion of S-Fe in acidic solution. Generally, organic compounds, especially those containing some hetero atoms in their structure are most commonly utilized as corrosion inhibitors for iron and steels in acidic media.^3–20^ The inhibition efficiency of these compounds relies on the concentration of the acidic solution, the nature of the metal or alloy used, the chemical composition of the additives and the capability to adsorb on the metal surface.

There are few studies on the inhibition of S-Fe corrosion in acidic solutions, because its use is concentrated in the Arabian Gulf region. Fawzy *et al.*^21^ used synthesized sodium *N*-dodecyl arginine compound as an efficient inhibitor for the corrosion of SABIC iron in acidic, alkaline, and neutral solutions. In previous studies expired antibacterial drugs,^22^ expired acyclovir and omeprazole drugs,^23^ and expired amoxicillin and cefuroxime drugs^24^ have been used as efficacious inhibitors for SABIC iron corrosion in 1.0 M hydrochloric acid solution. The high inhibition efficiency of these expired drugs was due to their strong adsorption on the S-Fe surface. A literature survey revealed that some polymer compounds have been applied to inhibit the corrosion of iron and steels in acidic solutions.^25–32^ For instance, polystyrene (PS) and polybutylene terephthalate (PBT) were previously used to inhibit the corrosion of steel in acidic solutions.^33,34^ The inhibition effect of these compounds depends on the chemical structure of the polymer and the presence of several active centers that enhance the adsorption process.

This study tested the use of two polymer compounds, namely polystyrene (PS) and polybutylene terephthalate (PBT), to retard the corrosion of S-Fe in 1 M HCl solution. The anticorrosive efficiency of PS and PBT compounds were measured by weight reduction (WR), galvanostatic polarization (GAP), potentiodynamic anodic polarization (PDAP), and electrochemical impedance spectroscopic (EIS) measurements. The thermodynamic functions for the activation and adsorption process were studied. Density functional theory (DFT) approaches were used to predict the corrosion inhibition performance. Theoretical quantum chemical calculations and Monte Carlo simulations were carried out on the studied polymers to correlate with the experimental findings.

## 2. Experimental

### 2.1. Materials

All the chemicals used, such as HCl, NaCl, and the tested two polymer compounds, namely PS and PBT, were supplied from Merck or Sigma-Aldrich. Double distilled water was used to prepare the solutions in all the investigations.

### 2.2. Technologies

SABIC iron (S-Fe) used in various technologies is produced by the Saudi Arabia Basic Industry Company (SABIC) with a purity of 99.99%. For weight reduction (WR), coupons with dimensions of 1 × 3.8 × 0.2 cm^3^ were used. The WR of S-Fe in mg was determined after an immersion time of 12 h in free 1 M HCl solutions and as well as in the presence of different concentrations of PS and PBT compounds. For the galvanostatic polarization (GAP), potentiodynamic anodic polarization (PDAP), and electrochemical impedance spectroscopic (EIS), cylindrical or galvanic polarization (GAP), potentiodynamic anodic polarization (PDAP), and electrochemical impedance spectroscopy (EIS) tests, a cylindrical S-Fe rod dipped in Araldites with an uncovered area of 0.38 cm^2^ was utilized. Prior to all experiments, the coupons or S-Fe rods were sanded with some grades of sand papers ranging from 300 to 1500 grades, after which they were cleaned with distilled water and acetone and finally dried by filter papers.

WR assays were done as formerly reported.^34,35^ GAP and PDAP technologies were performed utilizing a PGSTAT30 potentiostat/galvanostat in a conventional three-electrode cell with a platinum counter electrode (Pt), reference calomel electrode (RCE), and working electrode (S-Fe). In the electrochemical experiments, the electrode was immersed in the test solution until the steady state potential was attained after almost 40 min, and then the polarization started. The potential range for the PDP experiments was −1200 to +200 mV. For the GAP and PDAP tests, the scan rate was adjusted at 2.0 and 0.5 mV s^−1^, respectively. All the analyses were done at a constant temperature of 303 K in a temperature-controlled system. EIS was done in a frequency range of 100 kHz to 0.1 Hz with an amplitude of 4.0 mV from a peak-to-peak exploitation of AC signals in OCP.

### 2.3. Anticorrosive compounds

In this study, we used two polymer compounds, namely PS and PBT, as anticorrosive compounds, and these were purchased from Sigma Aldrich. The average molecular weights of PS and PBT were 35 000 and 90 000 g mol^−1^. The name, chemical structures, and IUPAC names are recorded in Table 1.

**Table 1:** Chemical structure and IUPAC names of the anticorrosion compounds

IUPAC names	Chemical structure	Name
Poly(1-phenylethene)	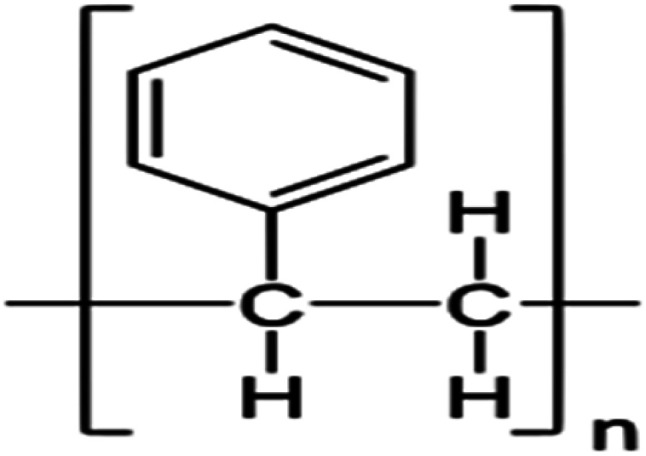	Polystyrene (PS)
Poly(oxy-1,4-butanediyloxycarbonyl-1,4-phenylenecarbonyl)	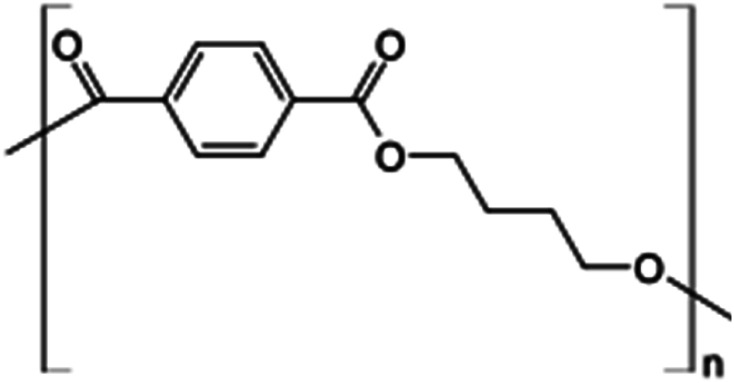	Polybutylene terephthalate (PBT)

### 2.4. Computational details

In this study, the two polymers were used as corrosion inhibitors for SABIC Fe. To conduct this computational study, the optimized structure geometries of both the dimer and the trimer of butylene terephthalate (D-BT and T-BT) and styrene (D-S and T-S) were found by density functional theory (DFT). The calculations were made using the B3LYP/6-31g(d,p) level of theory in aqueous media using a conductor-like polarizable continuum (CPCM) model.^36^ The energy of the highest occupied molecular orbital (*E*_HOMO_), energy of the lowest unoccupied molecular orbital (*E*_LUMO_), energy gap (Δ*E*), global hardness (*η* = Δ*E*/2), softness (*σ* = 1/*η*), back-donation (Δ*E*_b-d_), and dipole moment (*μ*) were all measured. The calculations were carried out using the Gaussian 09 code.^37^

### 2.5. Monte Carlo (MC) simulation

We carried out the MC simulation to study the interaction of the dimer polymer with the Fe (110) surface. The goal of this work was to determine the preferential adsorption of the dimer polymer on the iron surface. The simulation protocol was carried out on the Fe (110) plane with a five-layer thickness using the periodic boundary conditions. The Fe (110) plane was magnified to 15 × 15 supercells to provide a large surface area for interaction of the dimer inhibitor with the Fe (110) surface and a 25 Å vacuum was applied over the surface. The condensed-phase optimized molecular potentials for atomistic simulation studies (COMPASS) force field was utilized to search for the equilibrium configuration. The Ewald method was used to illustrate the electrostatic interactions with an accuracy of 1 × 10^−5^ kcal mol^−1^.^38^ The adsorption of the dimer inhibitor over Fe (110) was calculated by the following equation:

1 *E*_ads_ = *E*_Fe+dimer_ − *E*_dimer_ − *E*_Fe(110)_

where *E*_Fe+dimer_, *E*_dimer_, and *E*_Fe(110)_ are the energies of the dimer on Fe, energy of the dimer, and energy of the Fe (110), surface respectively.

## 3. Results and discussion

### 3.1. GAP tests

**Fig. 1:**
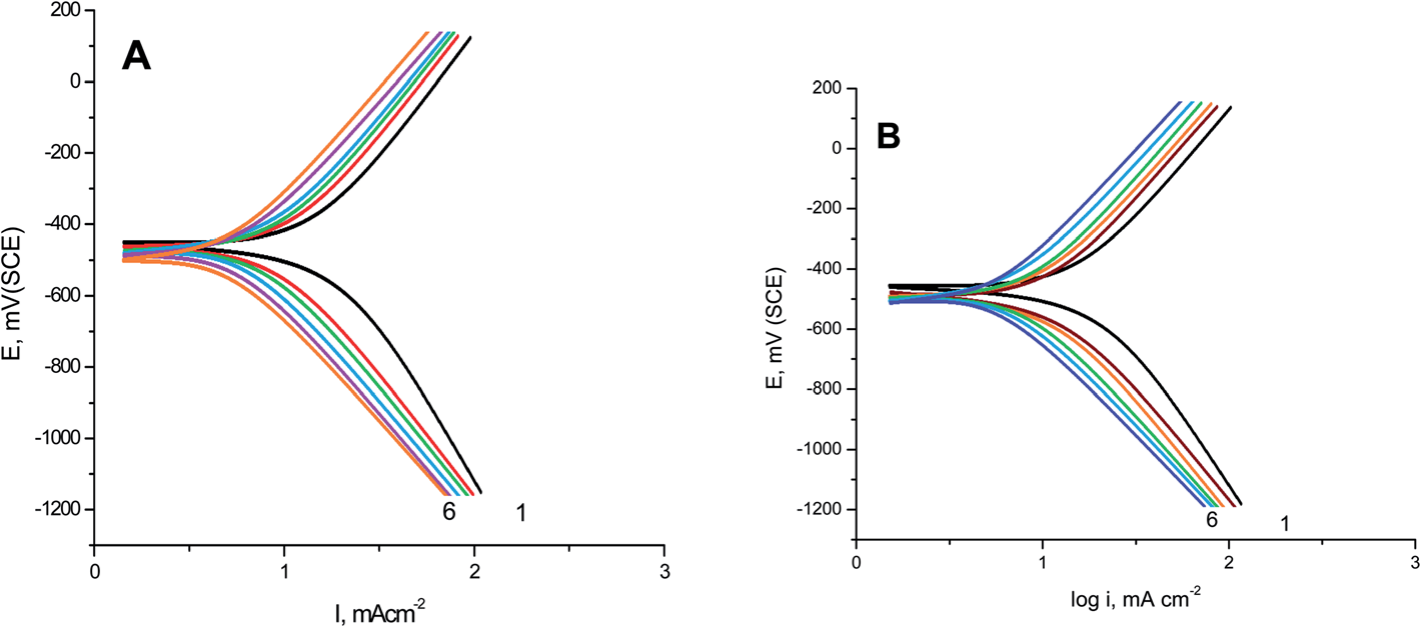
GAP curves for S-Fe corrosion in the free 1.0 M HCl solution and when containing different concentrations of (A) PS and (B) PBT at 300 K: (1) 0.0, (2) 100, (3) 200, (4) 300, (5) 400, (6) 500 mg l^−1^.

GAP curves of S-Fe in 1.0 M HCl solutions alone and in the presence of different concentrations of PS and PBT compounds are presented in Fig. 1A and B, respectively. Some corrosion functions, such as anodic (*β*_a_), cathodic (*β*_c_), and corrosion current density (*I*_corr._), which were determined from the intersection of the anodic and cathodic Tafel lines with the corrosion potential (*E*_corr._) and anticorrosion efficiency (%AE) were computed and are recorded in Table 2.

**Table 2:** Corrosion parameters obtained from the GAP measurements

Inh.	Inh. conc. (mg l^−1^)	−*E*_corr._ mV (SCE)	*β*_a_ (mV per decade)	−*β*_c_ (mV per decade)	*I*_corr._ (mA cm^−2^)	%AE_GAP_
—	0	475	132	180	8.960	—
PS	100	480	135	185	4.122	53.99
	200	482	140	189	2.951	67.06
	300	486	144	192	1.704	80.98
	400	488	148	196	0.851	90.50
	500	490	152	202	0.597	93.34
PBT	100	484	134	184	3.659	59.16
	200	487	142	188	2.329	74.01
	300	492	146	194	1.521	83.02
	400	495	150	200	0.805	91.02
	500	498	154	206	0.449	95.98

The percentage anticorrosion efficiency (%AE_GAP_) was calculated from the values of *I*_corr._ using the following equation:2
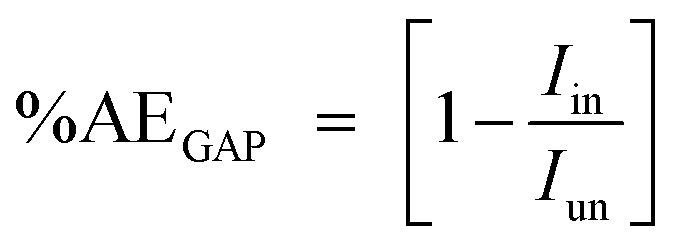
where *I*_in_ and *I*_un_ are the corrosion current densities in the blank 1.0 M Cl solutions and in the presence of different concentrations of the two polymer compounds (PS and PBT) ranging from 100 to 500 ppm.

It is evident that increasing the doses of polymer compounds in 1.0 M HCl solution hindered the anodic dissolution of iron and cathodic H_2_ evolution. In the beginning of the GAP curves, there were simultaneous anodic and cathodic curves. After this region, the potential increased with a positive trend (anodic polarization) and decreased with a negative trend (cathodic polarization). From Table 2, we note that the *β*_a_ and *β*_c_ were nearly constant, whereby the variation in *β*_a_ was about 20 mV per decade and the variation in *β*_c_ was about 22 mV per decade. These outcomes demonstrate the anticorrosive activity of the two tested polymer compounds occurred through adsorption on the surface of S-Fe according to a blocking adsorption mechanism. The adsorbed PS and PBT reduced the surface area available for anodic iron dissolution and cathodic H_2_ evolution reaction without altering the reaction mechanism. The constancy of the Tafel slopes demonstrated that the two polymer compounds (PS and PBT) could be classified as mixed inhibitors. Also, the *E*_corr._ values were nearly constant. The variations in the *E*_corr._ values were about 15 and 14 mV in the case of the PS and PBT compounds, respectively. These outcomes verified that the PS and PBT compounds were mixed inhibitors. Obviously, the *I*_corr._ values decreased significantly, while %AE_GAP_ was elevated at both PS and PBT concentrations. The maximum %AE_GAP_ values were 93.34% and 95.98% at 500 ppm of PS and PBT compounds. It is clear from Table 2 that the %AE_GAP_ for PBT in all the concentrations utilized was more than that of PS. This behavior is explained later in the mechanism of anticorrosive section.

### 3.2. EIS tests

**Fig. 2:**
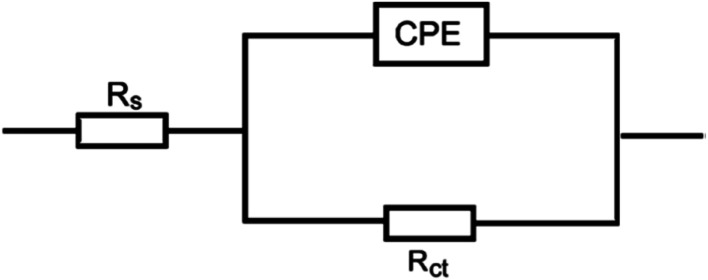
Electrochemical equivalent circuit applied to fit the EIS measurements.

**Fig. 3:**
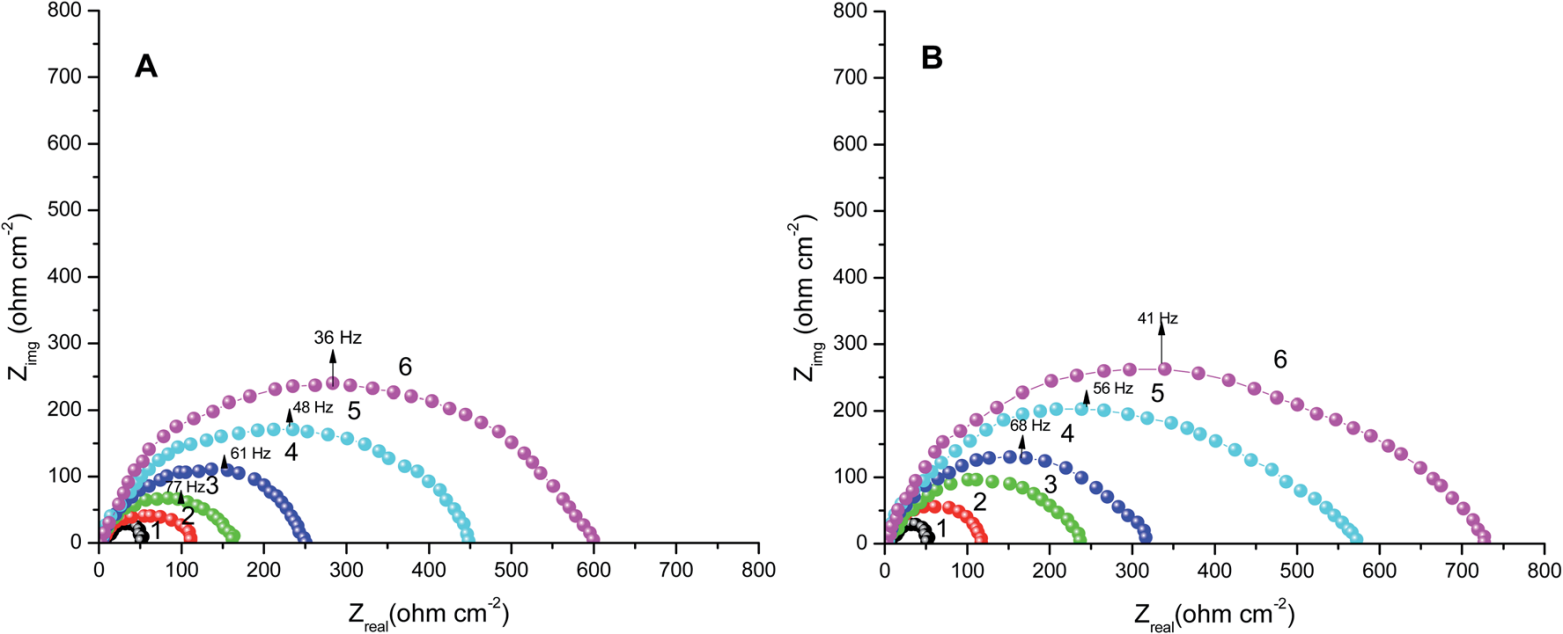
Nyquist plots for S-Fe corrosion in the blank 1.0 M HCl solution and when containing different concentrations of (A) PS and (B) PBT at 300 K: (1) 0.0, (2) 100, (3) 200, (4) 300, (5) 400, (6) 500 mg l^−1^.

The obtained results from the EIS tests can be stated in terms of the equivalent circuit (Fig. 2), which was applied in the previous embodiment of the SABIC Fe/acid interface.^24^Fig. 3A and B present Nyquist plots for the investigated steel in 1 M HCl with and without different doses of PS and PBT compounds. It is clear from these plots that the semicircle in the presence of PS and PBT is higher than that in the free acid solution which actually providing a protective behavior of the investigated compounds.^39^ This behavior is illustrated by the increased values of *R*_ct_ (Table 3), which could be due to the observed decrease in the *C*_dl_ values, especially at higher concentrations of additives used. This reveals the adsorption of the additive molecules on the surface of the corrosive CS, instead of the initially adsorbed water molecules.^40^ Also, the single semicircular capacitive ring observed in the free solution indicated a single charge-transfer process controlled the S-Fe corrosion process in this medium.^31^ The irregularity of the surface formed at the interface of the S-Fe solution was due to the heterogeneity and coarseness, which led to a decrease in the shape of the semicircle. The %AE_EIS_ values were computed according to the following equation:3
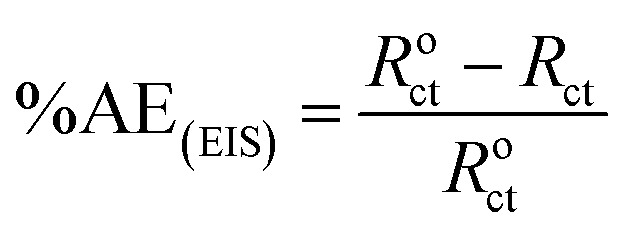
where *R*^o^_ct_ and *R*_ct_ are the charge-transfer resistance in the blank (1 M HCl) solution and in the presence of PS and PBT compounds, respectively. Whereas the double layer capacitance *C*_dl_ is given by the following equation:

4 *C*_dl_ = *Y*_0_(*W*_max_)^*n*−1^

where *W* is the angular frequency, *Y*_0_ and *n* are, respectively, the values of the CPE admittance and exponent.5
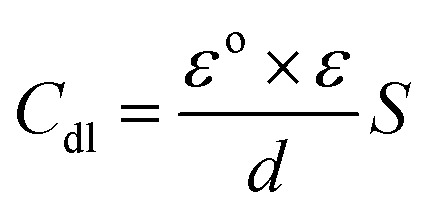
where *d* is the thickness of the adsorbed film, *ε*^o^ is the permittivity of air of the medium, *ε* is a dielectric constant, and *S* is the surface area.

**Table 3:** EIS data for S-Fe corrosion in free 1 M HCl solution and with different concentrations of PS and PBT compounds at 300 K

Inh.	Inh. conc. (mg l^−1^)	*R*_s_, ohm cm^−2^	*R*_ct_, ohm cm^−2^	*C*_dl_, (μF cm^−2^)	%AE
—	0	1.2	48	98	—
PS	100	1.3	115	45.4	58.26
	200	1.5	155	38.2	69.03
	300	1.6	245	30.8	80.41
	400	1.9	460	22.4	89.56
	500	2.1	605	17.4	92.07
PBT	100	1.4	120	42.6	60.00
	200	1.6	228	34.4	78.94
	300	1.8	312	28.6	84.61
	400	2.0	570	18.4	91.57
	500	2.2	725	12.6	93.37

**Fig. 4:**
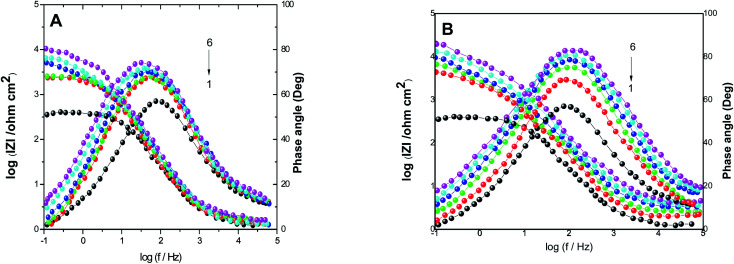
Bode plots for S-Fe corrosion in the blank 1.0 M HCl solution and when containing different concentrations of (A) PS and (B) PBT at 300 K: (1) 0.0, (2) 100, (3) 200, (4) 300, (5) 400 m (6) 500 mg l^−1^.

The electrochemical parameters, such as *R*_ct_, *C*_dl_, and AE_(EIS)_%, are recorded in Table 3. It is clear from this table that with the increase in the concentration of PS and PBT compounds, the *C*_dl_ values decreased due to the gradual replacement of water molecules by the adsorption of polymer compounds on the S-Fe surface, which formed a protective film on the S-Fe surface. For more evidence of the investigated inhibitors action against HCl corrosion, Bode and phase angle plots were made and are presented in Fig. 4A and B. As shown in the plots, the impedance modulus increased with the increase in the additive concentration at low frequencies, hence proving the adsorption of the PS and PBT compounds on the S-Fe surface, thus improving the inhibitory effect against HCl solution.^41^ Also, the appearance of a single peak in the phase angle diagrams illustrated the existence of a single time constant at the iron–solution interface.

### 3.3. PDAP measurements

**Fig. 5:**
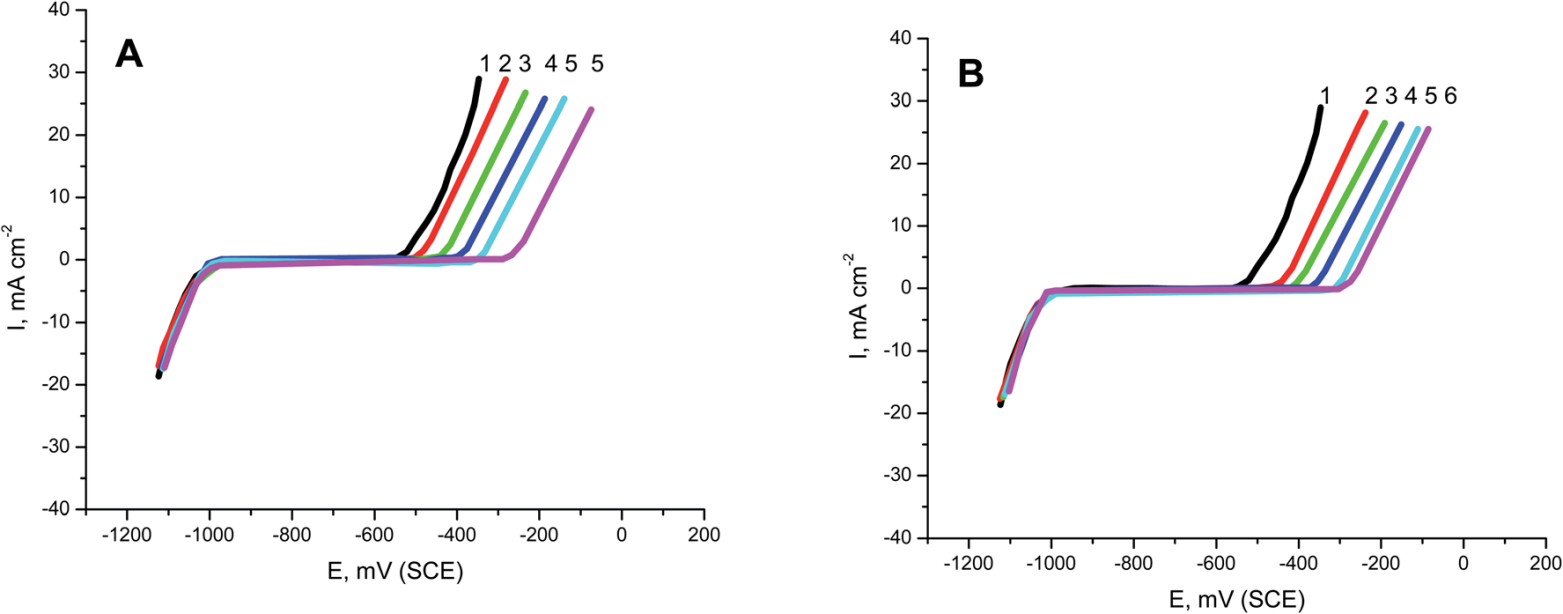
PDAP curves of S-Fe corrosion in the free 1.0 M HCl + 0.5 M NaCl solution and when containing different concentrations of (A) PS and (B) PBT at 300 K at a scan rate of 0.2 mV s^−1^: (1) 0.0, (2) 100, (3) 200, (4) 300, (5) 400, (6) 500 mg l^−1^.

PS and PBT compounds were examined as pitting corrosion inhibitors by utilizing PDAP measurements. Fig. 5A and B present the PDAP curves for S-Fe in 1.0 M HCl + 0.5 M NaCl solution and in the presence of some concentrations of PS and PBT compounds, respectively, at a scan rate of 0.2 mV s^−1^. NaCl solution was applied as a pitting corrosion factor. From this figure, it can be seen that there are no peaks in the anodic scan, which clarified the constancy of the passive film created on the iron surface. The current remained constant until a certain potential, when it rose rapidly due to the demolition of the passive film and formation of the pitting attack. This potential is defined as the pitting potential (*E*_pit._).^42,43^ With increasing the concentrations of PS and PBT, the values of *E*_pit._ moved to the noble (+) direction.

**Fig. 6:**
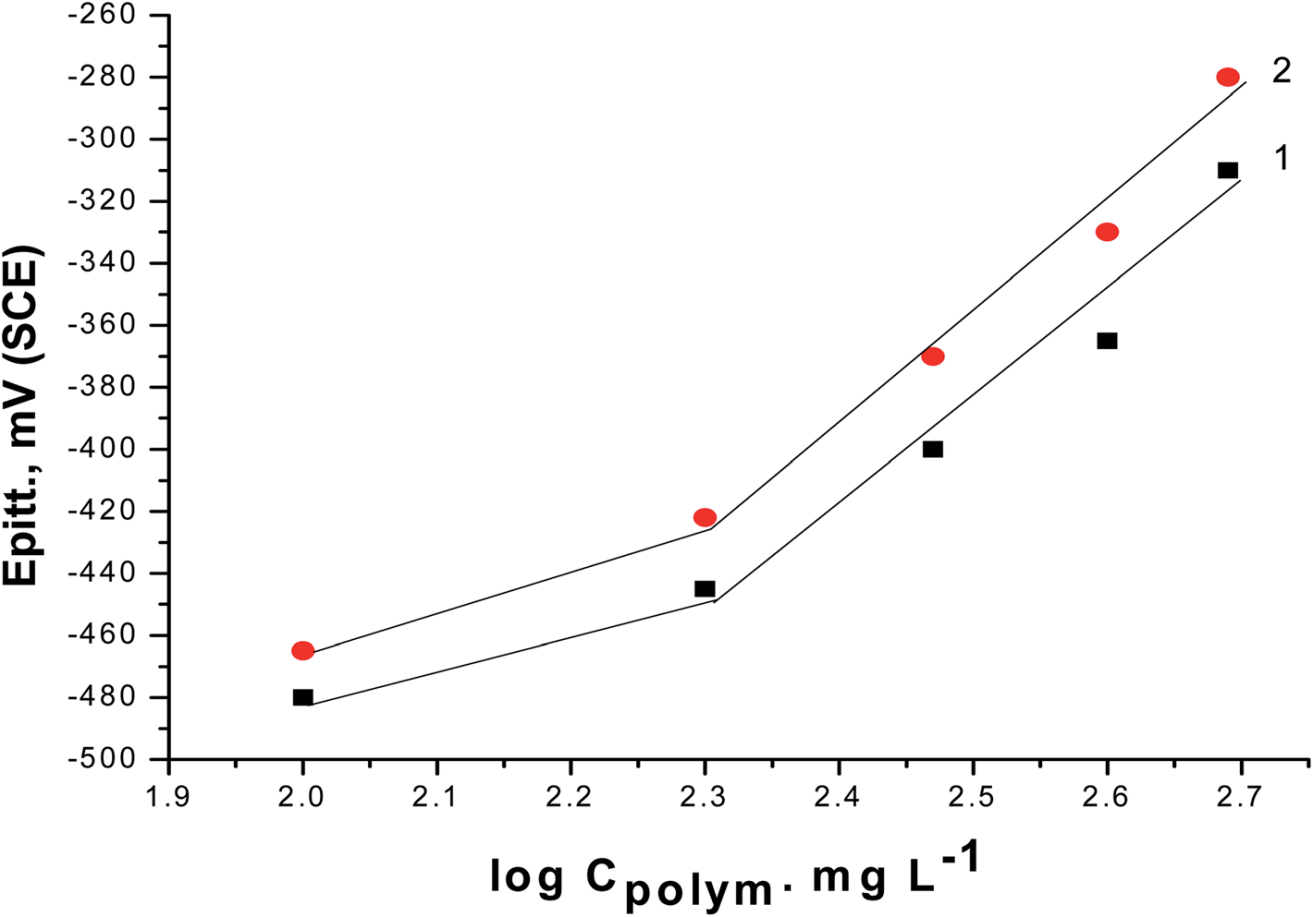
Relationship between *E*_pitt_ and the logarithmic concentrations of the two polymer compounds: (1) PS and (2) PBT.

Fig. 6 shows the relationship between the *E*_pit._ values and the logarithmic of the concentration of the two polymer compounds (PS and PBT). The same behavior was observed in the cases of the two polymers. Broken lines were obtained. At a lower concentricity (100 and 200 mg l^−1^) of PS and PBT, the shift in the *E*_pit._ values to the noble direction were low, but at a concentricity of more than 200 mg l^−1^, the *E*_pit._ values converted quickly to a noble trend according to the following equation:^44,45^

6 *E*_pit._ = *γ* + *β* log *C*_polymer_

where, *γ* and *β* are constants depending on the nature of the metal or alloy and the inhibitor used.

These outcomes confirmed that the presence of PS and PBT compounds increased the resistance to pitting corrosion. These compounds could thus be classified as pitting corrosion inhibitors. At all concentrations examined, the noble shift of *E*_pit._ (more resistance to pitting corrosion) for PBT compounds was more than for PS compounds. This completely agrees with the data acquired from the GAP and EIS measurements.

### 3.4. Weight reduction

#### 3.4.1. Impact of the polymer concentrations

The impact of different concentrations of PS and PBT compounds ranging from 100 to 500 mg l^−1^ on the dissolution of S-Fe in 1.0 M HCl solution was assessed by applying WR methods. The dissolution of S-Fe depended on the area of the coupons utilized and the immersion time. The corrosion rate (*K*_corr._) in mg cm^−1^ min^−1^ was computed from the subsequent equation:^43,44^7
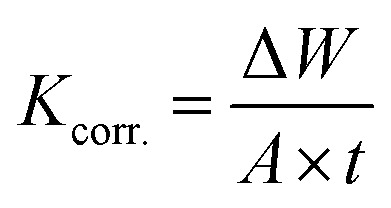
where Δ*W* in mg equal to (*W*_1_ − *W*_2_) and *W*_1_, *W*_2_ are the weight of S-Fe before and after incubation in the examined solutions, respectively.

The percentage anticorrosion efficiency (%AE_WR_) and surface coverage (*θ*) were determined from the values of *K*_corr._ using the following equations:^46,47^8
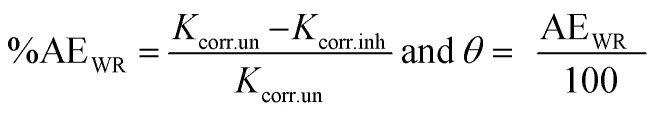
where *K*_corr.un_ and *K*_corr.inh_ are the corrosion rates in the blank 1.0 M HCl free solution and in the presence of two polymer compounds (PS and PBT).

The values of WR, *K*_corr._, *θ*, and %AE_WR_ in the cases of the PS and PBT compounds are listed in Tables 4 and 5, respectively. Cleary from this table, with increasing the concentration of the PS and PBT compounds, the WR decreased, which led to an increase in the %AE_WR_. These data confirmed the inhibitory power of the two polymer compounds was owing to their strong adsorption on the S-Fe surface. The %AE_WR_ reached 93.58% and 94.25% in the cases of 500 mg l^−1^ of the PS and PBT compounds, respectively. These data were roughly in agreement with the GAP and EIS results.

**Table 4:** Impact of increasing temperature on the corrosion functions acquired from WR measurements for the corrosion of S-Fe in blank 1.0 M HCl and in the presence of different concentrations of PS compounds

Temperature (K)	Conc. of PS (mg l^−1^)	WR (mg)	*K*_corr._ × 10^−4^ mg cm^−2^ h^−1^	*θ*	AE_(WR)_%
303	0.00	0.296	10.067	—	—
	100	0.133	4.523	0.551	55.07
	200	0.095	3.231	0.679	67.90
	300	0.059	2.001	0.801	80.12
	400	0.030	1.020	0.899	89.86
	500	0.019	0.646	0.936	93.58
313	0.00	0.389	13.229	—	—
	100	0.191	6.494	0.491	49.09
	200	0.147	4.999	0.622	62.21
	300	0.097	3.298	0.751	75.07
	400	0.058	1.972	0.851	85.09
	500	0.043	1.462	0.889	88.95
323	0.00	0.478	16.256	—	—
	100	0.267	9.081	0.441	44.14
	200	0.205	6.972	0.561	56.11
	300	0.138	4.693	0.711	71.13
	400	0.091	3.095	0.809	80.96
	500	0.076	2.585	0.841	84.09
333	0.00	0.562	19.113	—	—
	100	0.337	11.461	0.400	40.03
	200	0.275	9.355	0.511	51.05
	300	0.185	6.292	0.671	67.08
	400	0.135	4.591	0.760	75.98
	500	0.112	3.809	0.801	80.07

**Table 5:** Impact of increasing temperature on the corrosion parameters acquired from WR measurements for the corrosion of S-Fe in blank 1.0 M HCl and in the presence of different concentrations of PBT compounds

Temperature (K)	Conc. of PS (mg l^−1^)	WR (mg)	*K*_corr._ × 10^−4^ mg cm^−2^ h^−1^	*θ*	AE_(WR)_%
303	0.00	0.296	10.067	—	—
	100	0.121	4.115	0.591	59.12
	200	0.079	2.687	0.733	73.31
	300	0.053	1.802	0.821	82.09
	400	0.029	0.896	0.919	91.09
	500	0.017	0.578	0.943	94.25
313	0.00	0.389	13.229	—	—
	100	0.167	5.679	0.571	57.07
	200	0.121	4.115	0.689	68.89
	300	0.089	3.026	0.771	77.13
	400	0.054	1.836	0.861	86.12
	500	0.042	1.428	0.892	89.20
323	0.00	0.478	16.256	—	—
	100	0.219	7.448	0.542	54.18
	200	0.192	6.529	0.598	59.83
	300	0.162	5.509	0.661	66.11
	400	0.114	3.877	0.762	76.15
	500	0.100	3.401	0.791	79.08
323	0.00	0.562	19.113	—	—
	100	0.281	9.556	0.500	49.99
	200	0.253	8.604	0.550	54.98
	300	0.213	7.244	0.621	62.10
	400	0.162	5.510	0.712	71.17
	500	0.141	4.795	0.749	74.91

#### 3.4.2. Impact of elevated temperature

The effect of the elevated temperature from 300 K to 330 K on the corrosion data can be seen in the WR measurements of S-Fe in free 1.0 M HCl solution and when including different concentrations of PS and PBT compounds, and the results are tabulated in Tables 4 and 5. As the examined solution temperature increased from 303 K to 333 K, the values of WR and *K*_corr._ increased, while the values of *θ* and %AE_WR_ declined. This demonstrated the physical adsorption of PS and PBT compounds on the S-Fe surface *via* van der Waals interaction. High temperature may destroy the physical bonds, making the polymer compound desorb from the surface S-Fe.^48–50^

#### 3.4.3. Activation thermodynamic parameters

The values of the activation energy 
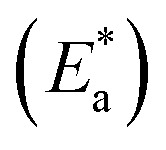
 for the dissolution of S-Fe in the blank 1.0 M HCl solutions and when adding specific concentrations ranging from 100 ppm to 500 ppm of PS and PBT compounds were evaluated by utilizing the Arrhenius equation as follows:^50,51^9
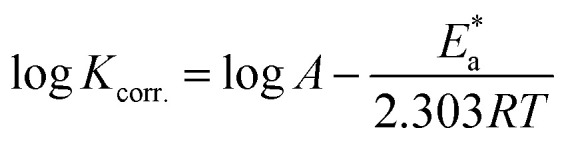
where *A*, *R*, and *T* represent the Arrhenius constant, gas constant, and absolute temperature, respectively.

**Fig. 7:**
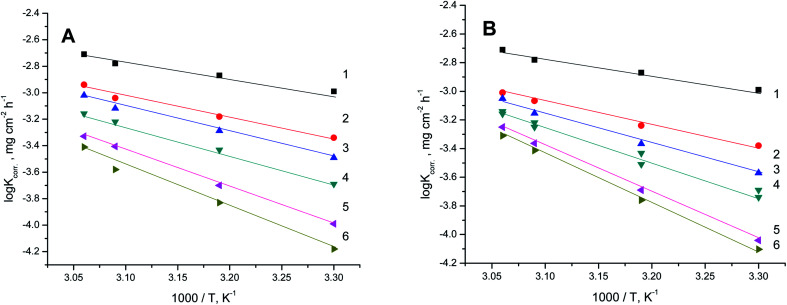
Plots of log *K*_corr._*versus* 1/*T* for S-Fe in the free 1 M HCl solution and in the presence of different concentrations of (A) PS and (B) PBT compounds. (1) 0.0, (2) 100, (3) 200, (4) 300, (5) 400, (6) 500 mg l^−1^.

Fig. 7A and B exhibit the plots of log *K*_corr._ and 1000/*T* for S-Fe in the 1 M HCl solution alone and in the presence of different concentrations of PS and PBT compounds. Straight lines were obtained with the linear regression coefficients nearly equal to one. The 
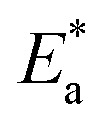
 values were assessed from the slope of the straight lines and are tabulated in Table 6. Obviously from the this table, it can be seen that the values of 
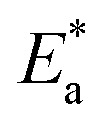
 in the presence of the two polymer compounds were greater than that observed in the blank 1.0 M HCl solution and the values of 
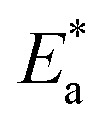
 increased with increasing the concentration of PS and PBT compounds. The increased activation energy of S-Fe corrosion indicated that the PS and PBT compounds acted as anticorrosion agents so as to delay the corrosion of S-Fe by the formation of a mass and charge-transfer barrier through them being adsorbed on the surface of S-Fe.

**Table 6:** Kinetics parameters for S-Fe in the blank 1 M HCl solution and in the presence of different concentrations of PS and PBT

Conc of inh. (ppm)	*E*_a_ kJ mol^−1^	ΔH* kJ mol^−1^	ΔS* J mol^−1^ K^−1^
1 M HCl	18.19	14.08	213.51
1 M HCl + 100 ppm PS	22.98	21.16	265.62
1 M HCl + 200 ppm PS	30.72	28.72	303.76
1 M HCl + 300 ppm PS	36.63	32.67	335.42
1 M HCl + 400 ppm PS	40.29	34.46	372.01
1 M HCl + 500 ppm PS	48.86	39.43	398.28
1 M HCl + 100 ppm PBT	26.81	25.68	295.92
1 M HCl + 200 ppm PBT	36.53	30.86	313.86
1 M HCl + 300 ppm PBT	45.95	44.33	356.80
1 M HCl + 400 ppm PBT	58.25	51.44	394.84
1 M HCl + 500 ppm PBT	68.92	60.29	436.26

The enthalpy (ΔH*) and entropy (ΔS*) of activation were determined by utilizing the following transition state equation:^50,51^10
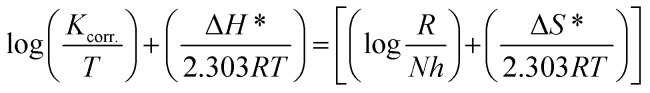
where *h* and *N* are Planck's constant and Avogadro's number, respectively.

**Fig. 8:**
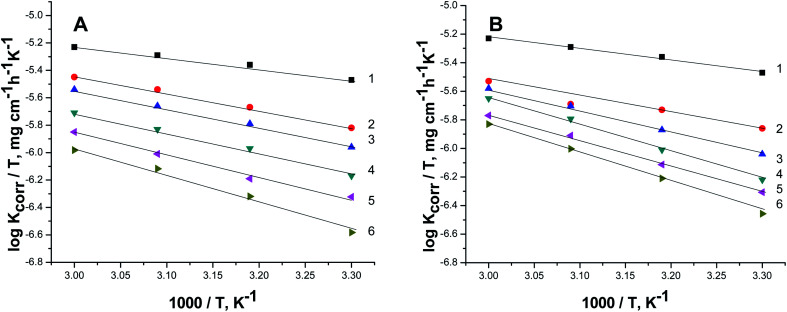
Plots of log *K*_corr._/*T versus* 1/*T* for S-Fe in the free 1 M HCl solution and in the presence of different concentrations of (A) PS and (B) PBT compounds: (1) 0.0, (2) 100, (3) 200, (4) 300, (5) 400, (6) 500 mg l^−1^.

Fig. 8A and B present the relationship between 
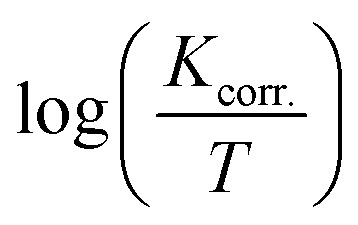
*versus*
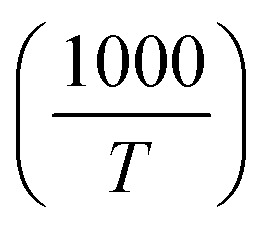
 for S-Fe in the blank 1 M HCl solution and in the presence of different concentrations of PS and PBT compounds. Straight lines were obtained. The ΔH* and ΔS* values were computed from the slopes and the intersections of the straight lines and are recorded in Table 6. It is evident from this table that the ΔH* values were positive in 1 M HCl solution alone and in the presence of different concentrations of the two polymer compounds (PS and PBT). This indicated the endothermic nature of the formation of the activated complexes during the corrosion process. Also, the values of ΔH* became more positive with increasing the concentrations of the PS and PBT, which made it more difficult for iron to corrode.^52^ The values of Δ*S*^o^ were negative and became less positive when the concentration of the two polymer compounds increased. This suggested that the compound activated in the rate-limiting step was involved in binding not disengagement. This clarifies that the activated molecules were in a less chaotic state than those in the initial stage.^53^ The anticorrosion efficiency of PS and PBT compounds with respect to the rise in the values of 
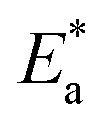
and ΔH*and the decline in the values of ΔS* followed the order: PBT > PS.

### 3.5. Adsorption and mechanism of the anticorrosion process

The anticorrosiveness of the two investigated polymer compounds (PS and PBT) on the corrosion of S-Fe in 1 M HCl solution principally relied on its adsorption at the S-Fe surface interface. On the whole, the adsorption operation involves an exchange process between the polymer compounds in the aqueous phase [polymer_(aq.)_] and the number of water molecules adsorbed on the S-Fe surface due to this equation:

11 Polymer_(aq.)_ + *α*H_2_O_(surf.)_ = polymer_(surf.)_ + *α*H_2_O_(aq.)_

where *α* is the amount of adsorbed water molecules that are replaced by one polymer molecule. The adsorption of PS and PBT on the S-Fe surface depended on several factors, including the concentration of the mineral acid and the polymer compounds used, the molar mass of the polymer, the temperature, the presence of certain active centers in the chemical structure of the polymer. The adsorption of the two polymer compounds on the surface of S-Fe diminished the rate of corrosion and elevated the anticorrosion effectiveness. To choose a suitable isotherm for this adsorption, the surface coverage values (*θ*) were applied to several isotherms. The results obtained confirmed that the preferable isotherm was the Langmuir isotherm, which can be given by:^54^12
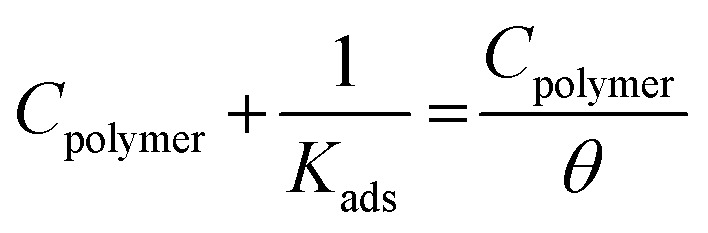
where *C*_polymer_ is the concentricity of the polymer used and *K*_ads_ is the equilibrium constant of adsorption.

**Fig. 9:**
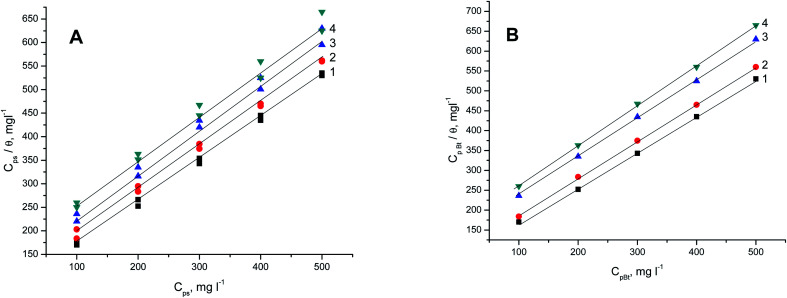
Langmuir adsorption isotherms for S-Fe in the blank 1 M HCl solution and in the presence of different concentrations of (A) PS and (B) PBT compounds at: (1) 303 K, (2) 313 K, (3) 323 K, (4) 333 K.

Fig. 9A and B present the Langmuir plots (*C*_polymer_ and *C*_polymer_/*θ*) for the adsorption of the two polymer compounds (PS and PBT) on the surface of S-Fe at altered temperatures ranging from 303 K to 333 K. A straight line with a slope of about one was obtained, which confirmed that the Langmuir isotherm was a suitable isotherm. This isotherm assumed there was zero interaction between the adsorbed species on the S-Fe surface. From the intercept of the Langmuir plots, we computed the values of *K*_ads_. Specifically, the values of *K*_ads_ for the PS compounds were computed from Fig. 9A and were (6.45, 5.71, 5.12, and 4.54) × 10^−3^ mol l^−1^ at temperatures of 303, 313, 323, and 333 K, respectively; while the values of *K*_ads_ for the PBT compounds were computed from Fig. 9B and were (6.25, 5.40, 4.82, and 4.04) × 10^−3^ mol l^−1^ at the temperatures of 303, 313, 323, and 333 K, respectively. The values of *K*_ads_ indicated that both PS and PBT on the surface of S-Fe were readily and cohesively adsorbed on the surface of S-Fe.

The values of free energy of adsorption (Δ*G*^o^_ads_) were determined from the values of *K*_ads_ according to the following equation:

13 55.5*K*_ads_ = exp(−Δ*G*^o^_ads_/*RT*)

The determined values of Δ*G*^o^_ads_ in the presence of PS compounds were −28.32, −27.14, −26.97, and −26.02 kJ mol^−1^ at temperatures of 303, 313, 323, and 333 K, respectively; while the values of Δ*G*^o^_ads_ in the presence of PBT compounds were −25.08, −24.42, −24.06, and −23.83 kJ mol^−1^ at temperatures of 303, 313, 323, and 333 K, respectively. It is evident that all the values of Δ*G*^o^_ads_ were negative, demonstrating that the adsorption of PS and PBT compounds on S-Fe was spontaneous. Vant's Hoff equation was applied to determine the enthalpy of adsorption Δ*H*^o^_ads_ according to the following equation:^55^14
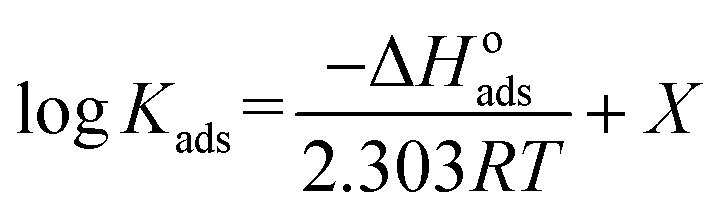
where *X* is a constant.

**Fig. 10:**
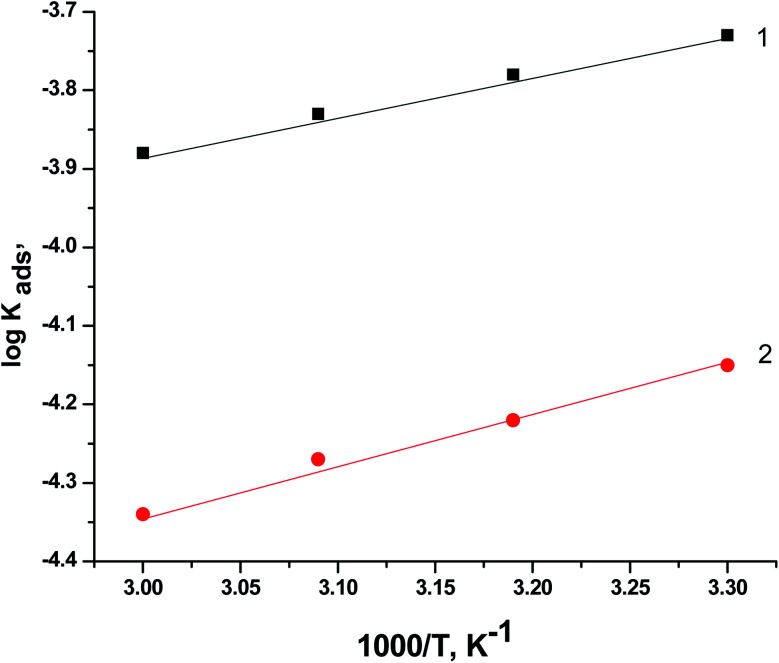
Plots of log *K*_ads_*versus* 1000/*T* for the corrosion of S-Fe in 1.0 M HCl solution in the presence of PS and PBT compounds at different temperatures: (1) PBT and (2) PS.

Fig. 10 displays the relationship between log *K*_ads_*vs.* 1/*T* (Van't Hoff plots) for PS and PBT adsorbed on the surface of S-Fe in 1.0 M HCl solution. Straight lines were obtained. From the slope of the straight lines, we could compute Δ*H*^o^_ads_. The values of Δ*H*^o^_ads_ were −19.88 and −21.12 kJ mol^−1^ for PS and PBT compounds, respectively. The negative mark of Δ*H*^o^_ads_ indicated that the adsorption of PS and PBT compounds on the S-Fe surface was exothermic.

The entropy of adsorption (Δ*S*_ads_) values were obtained from the following equation:15
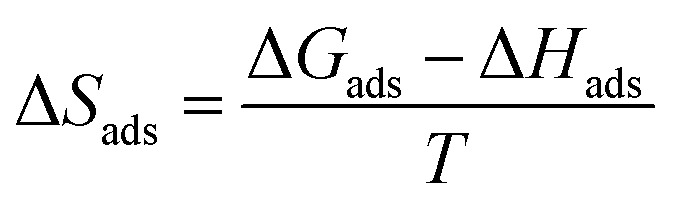


The computed values of Δ*S*_ads_ for PS compounds were −0.159, −0.086, −0.022, and −0.018 kJ mol^−1^ at temperatures of 303, 313, 323, and 333 K, respectively; while the values of Δ*S*_ads_ for PBT compounds were −0.013, −0.011, −0.009, and −0.008 kJ mol^−1^ at temperatures of 303, 313, 323, and 333 K, respectively.

The negative markings of Δ*S*_ads_ indicated that the chaos diminished upon transition from the reactant to the adsorbent surface. This demonstrates the adsorption intensity of the PS and PBT compounds on the surface of S-Fe. The values of the adsorption thermodynamic functions were consistent with the low values of the anticorrosion efficiency at elevated temperatures.

The corrosion functions and the anticorrosion efficiency gained from the various technologies confirmed that the %AE of PS compounds was greater than that of PBT compounds. These outcomes can be explicated on the basis of the molar mass of the polymer compound tested, where the molar mass of PBT is greater than that of PS. Thus PBT compounds will create a larger protective-layer covering on the surface than PS, giving a higher anticorrosion efficiency. This layer isolates the surface of the iron from the corrosive acidic solution and prevents contact of the S-Fe surface with the acidic solution.

The results of the PDAP measurements showed that the PS and PBT compounds had a high ability to resist the pitting corrosion by shifting the value of the pitting potential to the noble (+) direction. The resistance of pitting attack can be explicated by the competitive adsorption that occurred on the S-Fe surface between Cl^−^ ions and the PS and PBT compounds to reach the surface of S-Fe until the polymer compounds became dominant. This led to the formation of a protective layer on the surface of S-Fe, which impeded the pitting attack of chloride ions.

### 3.6. Computational study

**Fig. 11:**
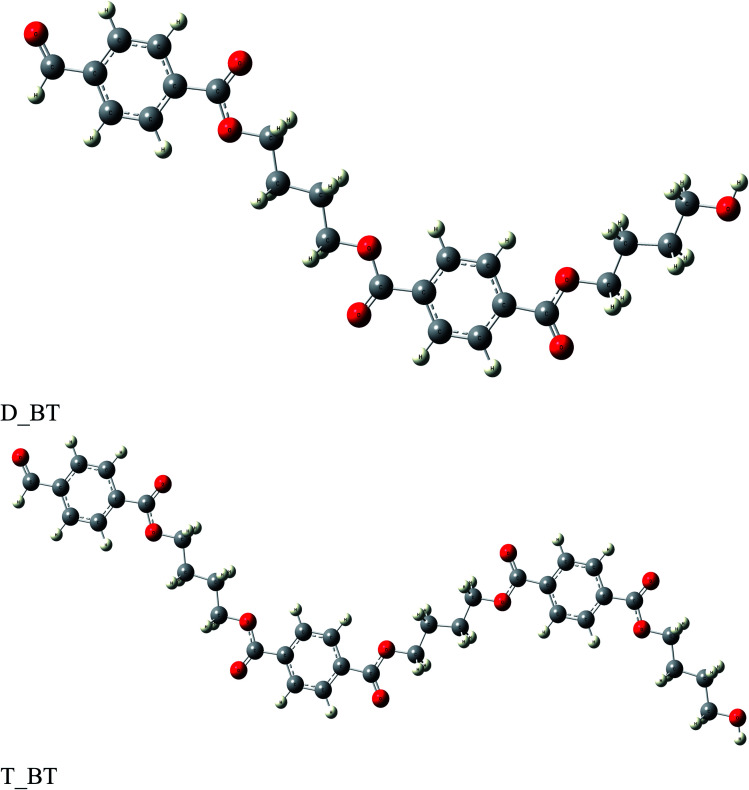
Optimized structure of di- and tri-butylene terephthalate.

**Fig. 12:**
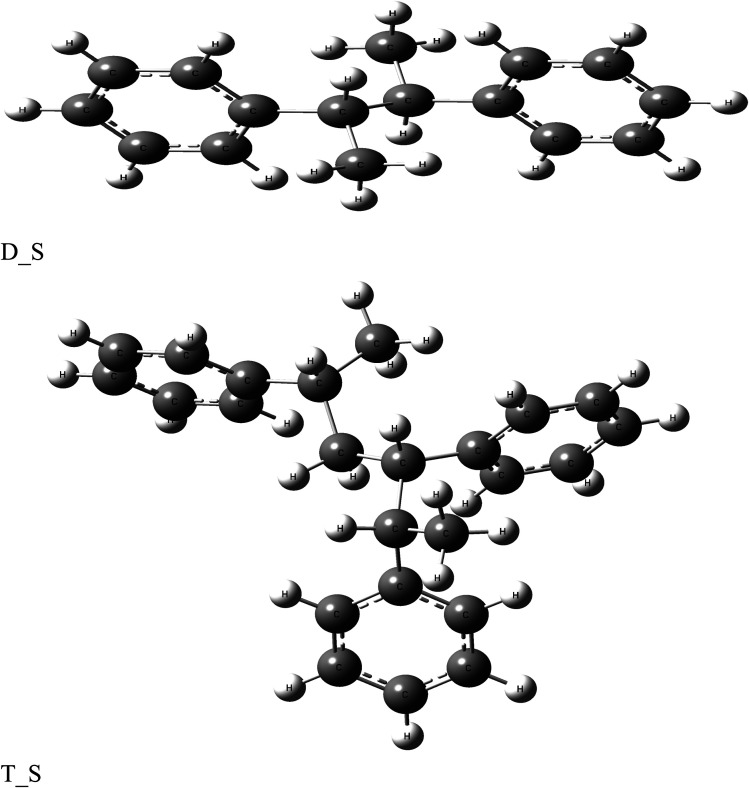
Optimized structure of di- and tri-styrene.

**Fig. 13:**
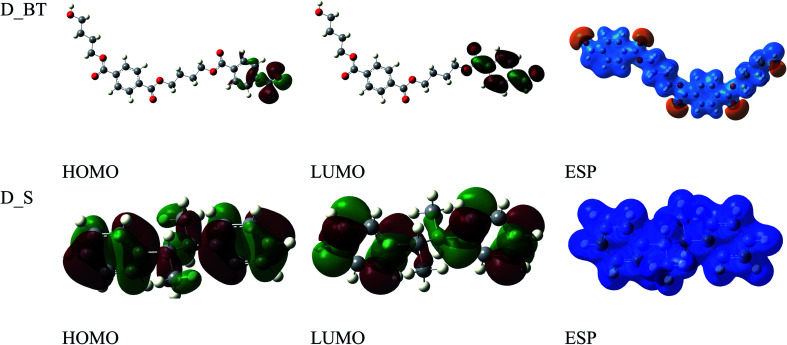
Frontier orbitals and ESP of di-butylene terephthalate and di-styrene.

The anticorrosion efficiency of the dimer and trimer of butylene terephthalate and styrene were investigated by finding the quantum parameters that correlated with the practical outcomes. The optimized geometries of the dimer and trimer of the two inhibitors are presented in Fig. 11 and 12. The optimizations were obtained by using the B3LYP level of theory. As shown in Table 7, most the quantum parameter values for the dimer and trimer of the butylene terephthalate inhibitor were the same, but there were slight changes between the dimer and trimer of the styrene inhibitor, so the frontier orbitals, molecular electrostatic potential, and adsorption of the polymer on the iron surface were investigated using the dimer molecules. The frontier molecular orbital (HOMO and LUMO) distributions could show the active sites that the inhibitors possess to interact with a metallic surface. As seen in Fig. 13, for the dimer of butylene terephthalate the HOMO was concentrated on the benzaldehyde moiety and LUMO in the terephthalate region, while the HOMO and LUMO distributions of the dimer of styrene were the same, indicating a high chance of electron transfer from the HOMO to the LUMO with bonding formation between the tested reagents.^54^ The high values of *E*_HOMO_ are likely to indicate a molecule's tendency to donate electrons to suitable acceptor molecules with empty molecular orbitals. The LUMO energy reflects a molecule's ability to receive electrons. So, with increasing the HOMO and lowering the LUMO energy levels, the inhibitor's ability to attach to the metal surface is improved. As shown in Table 7, the *E*_HOMO_ and *E*_LUMO_ of the dimer of butylene terephthalate were lower than the dimer of styrene. The energy gap (Δ*E*) is another parameter that is correlated with the anticorrosion efficiency. A lower energy gap value indicates a higher inhibition efficiency, because the energy of removing an electron from the highest occupied orbital is low;^55^ therefore, the higher reactivity of an inhibitor toward adsorption on metallic surfaces. The results obtained, as seen in Table 7, indicate that the dimer of butylene terephthalate had better anticorrosion efficiency than the dimer of styrene.

**Table 7:** Quantum parameters for di-, tri- PBT and PS calculated using B3LYP/6-31g(d,p) in the aqueous phase

	*E*_HOMO_ (eV)	*E*_LUMO_ (eV)	Δ*E* (eV)	*μ* (debye)	*σ* (eV)^−1^	*η* (eV)	Δ*E*_d-b_ (eV)
D_PS	−6.39	−0.17	6.22	0.0001	0.32	3.11	−0.78
T_PS	−6.39	−0.19	6.20	0.57	0.33	3.10	−0.77
D_PBT	−7.27	−2.35	4.92	2.41	0.41	2.46	−0.62
T_PBT	−7.27	−2.35	4.92	6.46	0.41	2.46	−0.62

The dipole moment (*μ*) is an index for predicting the direction of a corrosion inhibition process. The dipole moment is a measurement of polarity in a bond, which is linked to the electron distribution in a molecule. It is generally accepted that the adsorption of polar compounds with high dipole moments on the metal surface should result in improved anticorrosion efficiency. As shown in Table 7, the dipole moment of the dimer of butylene terephthalate was higher than that of the dimer of the styrene inhibitor, which showed a high symmetry of 0.0001 debye; therefore, the dimer of butylene terephthalate had a better anticorrosion efficiency than the dimer of styrene, which agreed with the experimental results.

The molecular electrostatic potential (ESP) is an important quantitative descriptor used to determine the reactive sites of electrophilic and nucleophilic attacks. As seen in Fig. 13, the negative electrostatic potential (red regions) for the dimer of butylene terephthalate was concentrated on the oxygen atom of C=O, indicating the acceptor sites. The positive electrostatic potential (blue regions) for the dimer of styrene was concentrated in all the molecules, which were thus associated with donor sites.

The softness (*σ*) and hardness (*η*) are called global reactivity descriptors. These parameters can be used to obtain the reactivity and stability of a molecule. The adsorption of polymer molecules on the S-Fe surface occur with a molecule that has a higher value of softness. Global hardness is a measure of the molecular resistance to electron cloud polarization or deformation due to minor chemical reaction disturbances. Soft molecules have a small energy gap but hard molecules have a higher energy gap.^56^ Generally, the inhibitor with the highest value of softness gives the highest anticorrosion efficiency.^57,58^ As presented in Table 7, the dimer of butylene terephthalate with a softness of 0.41 had the highest anticorrosion efficiency.

The back-donation of electrons (Δ*E*_b-d_) increases with the increase in anticorrosion efficiency. As shown in Table 7, this suggests the dimer of butylene terephthalate enhanced the back-donation when compared with the dimer of styrene.

### 3.7. MC simulation

**Fig. 14:**
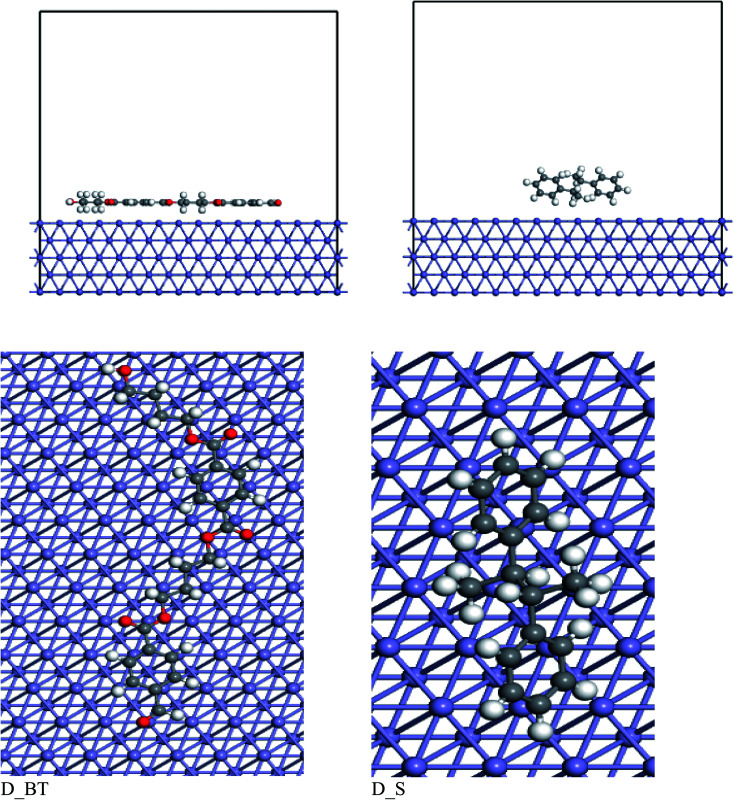
Top and side views for adsorption of the two dimers over Fe (110) surface.

The interaction assessment of the two dimers of butylene terephthalate and styrene on the Fe (110) surface was performed using Monte Carlo simulation.^59^Fig. 14 shows the most stable adsorption configurations of the polymer molecule on the Fe (110) surface. The dimer of butylene terephthalate was adsorbed in parallel on the Fe (110), but for the dimer of styrene, we found that the molecule was adsorbed by the two benzene rings tilted on the Fe (110) surface. When comparing the adsorption energy of the two inhibitors, as presented in Table 8, the dimer of butylene terephthalate had a higher adsorption energy than the dimer of styrene. These results confirmed the dimer of butylene terephthalate had a higher anticorrosion efficiency than the dimer of styrene. These results coincided with the experimental anticorrosion efficiencies.

**Table 8:** Adsorption energies of the two dimer inhibitors over the Fe (110) surface

System	Adsorption energy (kcal mol^−1^)
D_PS + Fe (110)	−82.95
D_PBT + Fe (110)	−231.06

## 4. Conclusions

The two polymer compounds (PS and PBT) acted as a good inhibitor for the corrosion of S-Fe in 1.0 M HCl solution. The anticorrosive efficiency rises with rising the concentrations of PS and PBT compounds. All the chemical, electrochemical and theoretical studies confirm that the anticorrosive efficiency of PBT is more than PS compound. The anticorrosive strength of these compounds was explicated by vigor spontaneous adsorption of these compounds on the S-Fe surface. The adsorption obeyed Langmuir isotherm. This theoretical study aims to compare the two polymer inhibitors based on quantum descriptors obtained by DFT. MC simulation was employed to determine the picture of the inhibitor molecule over the Fe (110) surface. The theoretical parameters are in agreement with experimental results.

## Conflicts of interest

There are no conflicts to declare.

## Supplementary Material
